# Accomodations for Delegates

**Published:** 1878-05

**Authors:** 


### EDITORIAL.

### American Medical Association.

The Committee of Arrangements in Buffalo are active in making preparation for the meeting of the Association, and for the accommodation of delegates and their families. They present for the information of the members of the Association the location and terms of the following Hotels.

| | | |
|---|---|---|
| Tifft House, 465 Main street, | $3.00 | per day. |
| Mansion House, cor. Main and Exchange | 2.50 | " |
| National Hotel, Exchange, opposite Central Depot, | 1.50 | " |
| Continental, Exchange, cor. Michigan street | 2.00 | " |
| Bonney's Hotel, Washington, cor Carroll street, | 1.50 | " |
| United States Hotel, Terrace, cor. of Pearl, | 2.00 | " |
| Broezel's Hotel, 127 East Seneca, | 2.00 | " |
| American Hotel, East Swan, cor. Ellicott, | 1.50 | " |

It is believed all guests will be thus amply provided for during the meeting.

